# Primary mucinous adenocarcinoma in a defunctionalized urinary bladder: a case report

**DOI:** 10.1186/1752-1947-3-9306

**Published:** 2009-11-30

**Authors:** Mary Taneous, Preetha Ramalingam, Donald G Mode, Jared G Heiner, Martha K Terris, Jeffrey R Lee

**Affiliations:** 1 Section of Pathology, Augusta Veterans Affairs Medical Center, 1 Freedom Way, Augusta, GA 30904, USA; 2 Department of Pathology, Medical College of Georgia, 1120 Fifteenth Street, Augusta, GA 30912, USA; 3 Section of Urology, Augusta Veterans Affairs Medical Center, 1 Freedom Way, Augusta, GA 30904, USA; 4 Section of Urology, Medical College of Georgia, 1120 Fifteenth Street, Augusta, GA 30912, USA

## Abstract

**Introduction:**

Malignancies are rare in defunctionalized bladders and are thought to arise from metaplasia secondary to chronic inflammation. Transitional cell and squamous cell carcinomas are the most common but there are three reported cases of mucinous adenocarcinoma.

**Case presentation:**

We report a 57-year-old Caucasian man presenting with penile discharge for 30 years following ileal conduit surgery for neurogenic bladder, and who was found to have primary mucinous adenocarcinoma of his defunctionalized bladder.

**Conclusion:**

Although urinary diversion without cystectomy is less common in current urologic practice, there are many patients with longstanding defunctionalized bladders. While there are no established surveillance protocols, defunctionalized bladder patients with urethral discharge should be evaluated.

## Introduction

Primary adenocarcinoma of the urinary bladder accounts for approximately 0.5-2% of all bladder cancers [1]. Patients generally present with hematuria, dysuria, suprapubic pain, and, less commonly, mucusuria. The histologic appearance of bladder adenocarcinoma can be enteric, signet ring, mucinous, clear cell, hepatoid and mixed types. The differential diagnosis includes glandular differentiation of transitional cell carcinoma and direct extension or metastatic spread of adenocarcinoma arising primarily from the colon, prostate, appendix or endometrium.

Predisposing factors for the development of primary adenocarcinoma of the bladder include schistosomiasis, exstrophy, persistent urachal remnants, and bladder augmentation by intestinal segments [1,2]. Concomitant cystitis glandularis occurs in the majority of cases, but is not considered a definitive precursor lesion since adenocarcinoma can develop in the absence of cystitis glandularis and only a small number of bladders with cystitis glandularis actually develop malignancies [2].

The potential for adenocarcinoma to develop in the defunctionalized bladder that is left in-situ at the time of urinary diversion is rare [3-5]. We describe the fifth case of primary mucinous adenocarcinoma arising in a longstanding defunctionalized bladder.

## Case presentation

A 57-year-old Caucasian man presented with profuse blood-streaked mucous drainage from his penis. Past medical history was significant for T12 spinal cord injury secondary to a motor vehicle accident in 1971. Due to urinary incontinence exacerbating his multiple decubitus ulcers and severe bilateral vesico-ureteral reflux, the patient underwent ileal conduit urinary diversion in 1975 with his bladder left in-situ.

**Figure 1:**
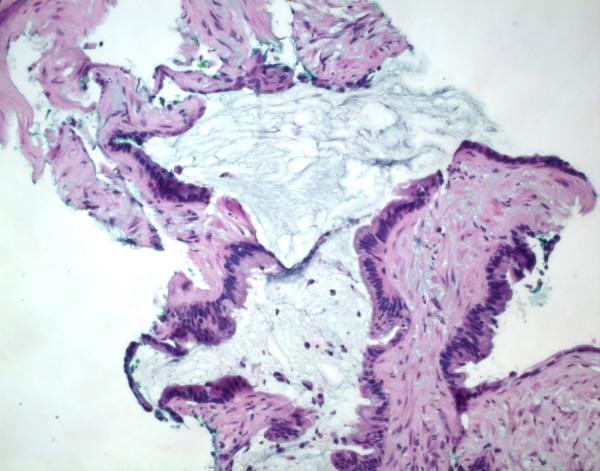
**Hematoxylin and eosin stain demonstrating mucinous adenocarcinoma within prostatic needle core biopsy (×100)**.

On initial evaluation for his penile discharge, he was found to have a right prostatic nodule on physical examination with a normal prostate specific antigen level of 1.5 ng/mL. Prostate biopsies showed mucinous adenocarcinoma in six of eight cores with large collections of mucin dissecting the prostatic parenchyma. Banal appearing columnar cells lined these mucous lakes, and nests of these cells were seen floating in the mucin (Figure 1). Immunohistochemical evaluation revealed that neoplastic cells were positive for carcinoembryonic antigen, cytokeratin-7, cytokeratin-20 and were negative for prostate specific antigen and prostatic acid phosphatase.

**Figure 2:**
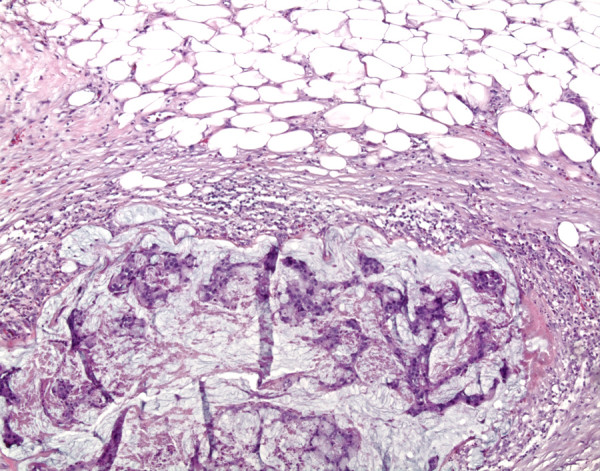
**Hematoxylin and eosin stain demonstrating mucinous adenocarcinoma extending into perivesicular fat (×100)**.

**Figure 3:**
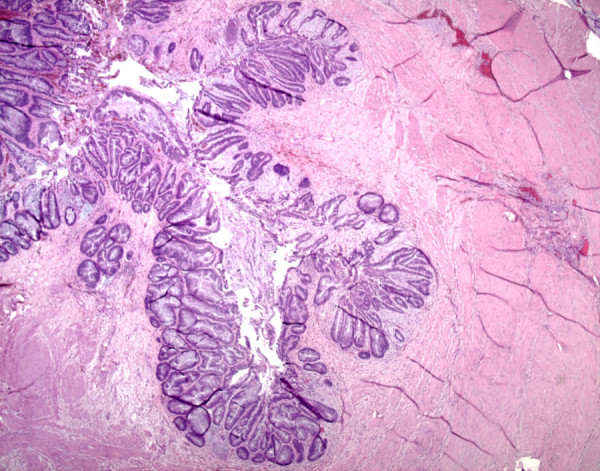
**Hematoxylin and eosin stain demonstrating extension of cystitis glandularis with intestinal metaplasia into distal remnant ureter (×25)**.

Further workup included colonoscopy showing multiple adenomatous polyps were identified without invasive malignancy and cystoscopic evaluations. Cystoscopy revealed a massive amount of mucoid material within the bladder associated with a mass. Bladder washings and biopsies revealed copious amounts of mucin with associated malignant nests and single cells also consistent with mucinous adenocarcinoma. Staging evaluation revealed no evidence of metastatic disease but at the time of radical cystectomy, periaortic lymphadenopathy and a locally extensive tumor mass were identified. Palliative cystoprostatectomy was performed. The cystoprostatectomy specimen demonstrated a circumferential mass with glistening mucosal surface that involved the posterior and lateral bladder surfaces, trigone, bladder dome, and invaded the prostatic parenchyma. On histologic examination, lakes of infiltrating mucin dissected through the muscularis propria of the bladder and into the perivesicular fat (Figure 2). The adjacent mucosa showed extensive glandular metaplasia with areas of high-grade dysplasia. The metaplastic glandular changes extended into the remnant distal ureters (Figure 3).

Postoperatively, the patient underwent adjuvant radiation and chemotherapy, but developed metastatic intraperitoneal and pulmonary masses 18 months following diagnosis. He expired 29 months after diagnosis.

## Discussion

Historically, supravesical intestinal urinary diversion without cystectomy was a common procedure employed to control the sequelae of neurogenic bladder [4]. Advances in medical management, intermittent catheterization, and augmentation procedures have significantly decreased, but not completely eliminated, the need for intestinal urinary diversion in neurogenic bladder patients.

The most common complaint of patients with defunctionalized bladders is urethral discharge, occurring in approximately 30% of patients [4]. Patients with intact sensation will often complain of pain. Hematuria and infection, particularly pyocystis and urosepsis, are the most frequent indications for additional surgical intervention. There is general awareness of the increased risk of malignancy in the intestinal segments used in urinary diversion and both the intestine and urothelium following intestinal augmentation of the bladder. The potential for malignancy in the bladder that is left in-situ and defunctionalized by urinary diversion is less well recognized [5]. When malignancy develops in the setting of a defunctionalized bladder, tumor types consist predominantly of squamous cell carcinoma and transitional carcinoma. Only six prior cases of primary adenocarcinoma in a defunctionalized, non-augmented bladder have been described: one signet ring adenocarcinoma [6], one enteric adenocarcinoma [7], and four mucinous adenocarcinomas [8-10].

Of the six adenocarcinomas in defunctionalized bladders, one patient had bladder dysfunction associated with prune belly syndrome [8], and five patients had neurogenic bladder dysfunction (three secondary to traumatic spinal cord injury [7-9], one patient with meningomyelocele [9], and one with imperforate anus and likely associated sacral abnormalities [10]). Our patient also had neurogenic bladder dysfunction secondary to traumatic spinal cord injury. Combining our patient with the three prior reported cases of mucinous adenocarcinoma in defunctionalized, non-augmented bladders, four were associated with cystitis glandularis [7-9] and one had intestinal metaplasia. The time interval from supravesical diversion to development of mucinous adenocarcinoma in these five patients ranged from 8 to 39 years (average 23.9 years). In contrast, squamous cell carcinoma, transitional cell carcinoma, and non-mucinous adenocarcinomas developed in defunctionalized bladders an average of 5 years after supravesical diversion [9]. In 80% (four of five cases), the patients presented with mucoid or bloody urethral discharge; the remaining case presented with urosepsis. Four of the five mucinous adenocarcinomas in defunctionalized bladders have been highly aggressive, with either extensive disease at initial surgery or early recurrence/progression [7-9].

## Conclusion

As large numbers of spinal cord injury patients with supravesical intestinal diversions approach 20 to 40 years postoperatively, malignancies in the defunctionalized bladders of these patients may become more frequent. There are currently no guidelines for bladder screening in these patients who appear to be at risk. Yap et al. recommend close follow-up with cystoscopy and bladder wash cytology on an annual or biennial basis in patients with a defunctionalized bladder [10]. The development of mucoid urethral discharge or bleeding in patients with long-term defunctionalized bladders should raise the suspicion of malignancy and prompt evaluation. When performing urinary diversion for neurogenic bladder, prophylactic simple cystectomy should be considered to eliminate the possibility of future development of bladder carcinoma.

## Consent

Written informed consent was obtained from the patient for publication of this case report and any accompanying images. A copy of the written consent is available for review by the Editor-in-Chief of this journal.

## Competing interests

The authors declare that they have no competing interests.

## Authors' contributions

MT, PR, and JRL performed the sections, stains, and histological examination and analysis of the bladder specimen, and were major contributors in assessing the pathology literature and writing the histologic description and theoretical pathogenesis portion of the manuscript. DGM, MKT and JGH performed the surgical procedures and were major contributors in the identification of past reported cases and writing the urological and historical perspectives of the manuscript. All authors read and approved the final manuscript.
